# The Mosaic Mutants of Cucumber: A Method to Produce Knock-Downs of Mitochondrial Transcripts

**DOI:** 10.1534/g3.115.017053

**Published:** 2015-04-14

**Authors:** Angel R. Del Valle-Echevarria, Agnieszka Kiełkowska, Grzegorz Bartoszewski, Michael J. Havey

**Affiliations:** * Department of Horticulture, University of Wisconsin, Madison, Wisconsin 53706; † Faculty of Horticulture, Agricultural University of Krakow, Al. 29 Listopada 54, 31-425 Krakow, Poland; ‡ Department of Plant Genetics, Breeding and Biotechnology, Faculty of Horticulture, Biotechnology and Landscape Architecture, Warsaw University of Life Sciences, ul. Nowoursynowska 159, 02-776 Warsaw, Poland; § USDA Agricultural Research Service, University of Wisconsin, Madison, Wisconsin 53706

**Keywords:** plant mitochondria, mitochondrial mutant, mitochondrial transcript, *Cucumis sativus*

## Abstract

Cytoplasmic effects on plant performance are well-documented and result from the intimate interaction between organellar and nuclear gene products. In plants, deletions, mutations, or chimerism of mitochondrial genes are often associated with deleterious phenotypes, as well as economically important traits such as cytoplasmic male sterility used to produce hybrid seed. Presently, genetic analyses of mitochondrial function and nuclear interactions are limited because there is no method to efficiently produce mitochondrial mutants. Cucumber (*Cucumis sativus* L.) possesses unique attributes useful for organellar genetics, including differential transmission of the three plant genomes (maternal for plastid, paternal for mitochondrial, and bi-parental for nuclear), a relatively large mitochondrial DNA in which recombination among repetitive motifs produces rearrangements, and the existence of strongly mosaic (MSC) paternally transmitted phenotypes that appear after passage of wild-type plants through cell cultures and possess unique rearrangements in the mitochondrial DNA. We sequenced the mitochondrial DNA from three independently produced MSC lines and revealed under-represented regions and reduced transcription of mitochondrial genes carried in these regions relative to the wild-type parental line. Mass spectrometry and Western blots did not corroborate transcriptional differences in the mitochondrial proteome of the MSC mutant lines, indicating that post-transcriptional events, such as protein longevity, may compensate for reduced transcription in MSC mitochondria. Our results support cucumber as a model system to produce transcriptional “knock-downs” of mitochondrial genes useful to study mitochondrial responses and nuclear interactions important for plant performance.

The plant cytoplasm carries two organelles, the plastids and mitochondria, each with their own DNA. The plant-organellar genomes encode a subset of the tRNAs, ribosomal subunits, and proteins required for organellar gene expression and function (Unseld *et al.* 1997; Mackenzie and McIntosh 1999; Notsu *et al.* 2002; Handa 2003; Ogihara *et al.* 2005; Sugiyama *et al.* 2005; Allen *et al.* 2007). In addition, thousands of nuclear-encoded proteins are required for functional organelles (Emanuelsson *et al.* 2000; Giegé *et al.* 2005). The intimate interactions between organelles and the nucleus are important components of plant performance (Kihira 1982), cytoplasmic male sterility (CMS) used to produce hybrid seed (Hanson 1991; Bentolila *et al.* 2002), and abiotic-stress tolerances (Atkin and Macherel 2009; Vanlerberghe *et al.* 2009; Gill and Tuteja 2010; Miller *et al.* 2010; Juszczuk *et al.* 2012).

The sizes of plant mitochondrial DNA (mtDNA) vary greatly, from approximately 208 kb (Palmer and Herbon 1987) to well over 11 megabases (Sloan *et al.* 2012). These enormous size differences are not due to greater coding capacity of the DNA, but rather are largely due to the accumulation of repetitive DNA. Recombination among these repetitive motifs allows for intramolecular or intermolecular recombination that give rise to rearranged mtDNA that can exist as relatively low-copy molecules (sublimons) (Fauron *et al.* 1995; Lilly and Havey 2001; Abdelnoor *et al.* 2003, 2006; Bartoszewski *et al.* 2004b). Many mitochondrial sublimons can exist in varying proportions among individual plants or lines (Mackenzie *et al.* 1994; Fauron *et al.* 1995; Lilly and Havey 2001; Abdelnoor *et al.* 2003, 2006; Bartoszewski *et al.* 2004b; Woloszynska 2010). Less prevalent mtDNA sublimons may increase in frequency relative to the predominant molecule, referred to as substoichiometric shifting (Mackenzie *et al.* 1994; Woloszynska 2010; Shedge *et al.* 2007). Substoichiometric shifting can be under nuclear control (Mackenzie *et al.* 1994; Abdelnoor *et al.* 2003; Shedge *et al.* 2007; Arrieta-Montiel *et al.* 2009) or may occur after passage through cell cultures (Hartmann *et al.* 1994; Malepszy *et al.* 1996; Gutierres *et al.* 1997). As a result, unique phenotypes associated with mitochondrial mutations or rearrangements may appear as the prevalence of specific mtDNAs changes.

Mutations in the mtDNA are useful to provide insights about the basic biology of the organelle as well as nuclear responses. Most mitochondrial mutations are deleterious; for example, the *chm*-induced chlorotic phenotypes of *Arabidopsis* (Sakamoto *et al.* 1996), nonchromosomal stripe (NCS) of maize (Newton 1993), and mosaic (MSC) cucumber (Malepszy *et al.* 1996; Lilly *et al.* 2001) are associated with low germination, reduced fitness, and distinctive pale sectored lines or regions on leaves. The *chm*-associated phenotypes of *Arabidopsis* are associated with substoichometric shifting of rarer mtDNAs (Martínez-Zapater *et al.* 1992). The *chm* locus has been cloned and encodes a MutS HOMOLOG1 (MSH1) protein (Abdelnoor *et al.* 2003); mutations in MSH1 result in lower stability of the organellar genomes to produce variant phenotypes (Xu *et al.* 2012). Various NCS mutants possess deletions in mitochondrial genes affecting translation (NCS3 and NCS4) or in complexes I (NCS2) and IV (NCS5 and NCS6) of the electron transport chain (Karpova and Newton 1999; Gu *et al.* 1993; Jiao *et al.* 2005). The MSC phenotypes of cucumber appear after passage through cell cultures (Bartoszewski *et al.* 2007), possess deleted regions associated with mtDNA rearrangements (Lilly *et al.* 2001), and suffer energy deficiency due to instability of complex I of the electron transport chain (Juszczuk and Rychter 2009).

There is presently no efficient method to produce mutants of mitochondrial genes. Challenges to generating and selecting mitochondrial mutants include multiple mitochondria per cell, each with multiples copies of mtDNA (Mileshina *et al.* 2011; Colas Des Francs-Small and Small 2014). Colas Des Francs-Small and Small (2014) proposed that mutating genes for nuclear-encoded, mitochondrially targeted proteins might be an effective way to produce “indirect mitochondrial mutants.” Justification for this approach is that certain mitochondrial mutations would likely be lethal, making them difficult or impossible to isolate.

Cucumber has been proposed as a model plant for organellar genetics (Bartoszewski *et al.* 2007). The three genomes of cucumber show differential transmission (bi-parental for the nucleus, maternal for chloroplast, and paternal for mitochondria), allowing for separation of organellar effects by reciprocal crossing (Havey *et al.* 1998). Uninucleate microspores of cucumber possess relatively few huge mitochondria (Abreu *et al.* 1982). As microspores mature to bi-cellular pollen, the mitochondria divide and resume normal shape, size, and numbers (Abreu *et al.* 1982). The formation of relatively few huge mitochondria during microsporogenesis likely creates a bottleneck reducing the diversity of mtDNAs transferred via the male gametophyte to progenies. This bottleneck may contribute to the sorting of mtDNAs revealed during transmission studies (Lilly *et al.* 2001). Cucumber also has one of the largest mtDNAs at 1685 kb (Alverson *et al.* 2011), more than four-times larger than other plants in the family Cucurbitaceae, such as watermelon (*Citrullus lanatus*) at 379 kb (Alverson *et al.* 2010). This significant expansion in mtDNA size is due, in part, to the accumulation of short repetitive DNA motifs throughout the molecule (Lilly and Havey 2001; Bartoszewski *et al.* 2004a, Alverson *et al.* 2011). Intragenomic or intergenomic recombination among these repetitive regions produces rearrangements in the mtDNAs (Lilly *et al.* 2001; Bartoszewski *et al.* 2004a; Alverson *et al.* 2011). Finally, there exist paternally transmitted MSC phenotypes of cucumber (Malepszy *et al.* 1996; Lilly *et al.* 2001; Bartoszewski *et al.* 2004b). Passage of the highly inbred (>S_18_) wild-type line "B" through cell cultures followed by regeneration of plants produces progenies with the MSC phenotypes (Malepszy *et al.* 1996, Bartoszewski *et al.* 2007). These phenotypes are associated with rearrangements in the mtDNA, show altered mitochondrial gene expression, and are not due to imprinting of paternal alleles (Lilly *et al.* 2001; Bartoszewski *et al.* 2004b). Independently produced MSC lines, all derived from highly inbred B, have been described (Ładyżyński *et al.* 2002) and can be distinguished by visual phenotypes (Malepszy *et al.* 1996) and unique rearrangements in their mtDNAs (Bartoszewski *et al.* 2004b, 2007). Independently derived MSC lines may trace back to different mitochondrial sublimons in inbred "B" that become more prevalent during cell culture (Hauschner *et al.* 1998; Hartmann *et al.* 2000), or passage through cell culture could produce *de novo* rearrangements in the cucumber mitochondrial genome. One advantage of cucumber, as compared to *chm*-associated rearrangements of *Arabidopsis* (Shedge *et al.* 2007) or the NCS mutants (Newton and Coe 1986) and P2 line of maize (Kuzmin *et al.* 2005), is that different MSC lines can be produced and maintained in the same highly inbred background without manipulation of nuclear alleles and their mitochondrial origin can be confirmed by paternal transmission (Bartoszewski *et al.* 2007).

In this study, we sequenced the mtDNAs of three MSC mutants (MSC3, MSC12, and MSC16) independently produced from the wild-type, highly inbred line B and identified regions of lower coverage relative to B. Our results support cucumber as a model plant to produce independent mitochondrial mutants in a highly inbred nuclear background.

## Materials and Methods

### Plant material and growing conditions

**Figure 1:**
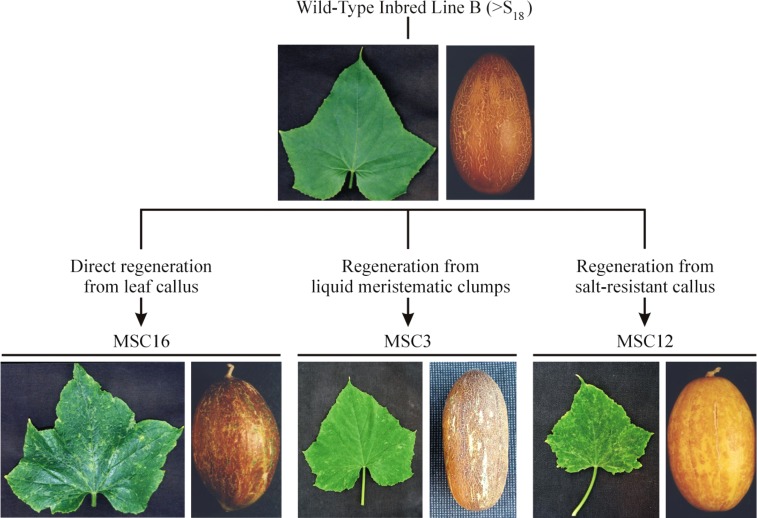
Origins and phenotypes of the mosaic (MSC) mutants of cucumber used in this study. The highly inbred line B was passed through cell cultures and regenerated plants were self-pollinated to produce the MSC lines (Bartoszewski *et al.* 2007).

The origins and phenotypes of wild-type inbred B and MSC lines 3, 12, and 16 have been previously described (Malepszy *et al.* 1996; Lilly *et al.* 2001; Bartoszewski *et al.* 2007). Cucumber seeds from inbred line B, MSC3, MSC12, and MSC16 were germinated in vermiculite at 30° in greenhouses on the UW-Madison campus. Plants sampled for analyses of their genome, transcriptome, or proteome were visually similar and true-to-type for either their MSC or their wild-type phenotypes (Figure 1).

### Total DNA and RNA isolation

Plants for genomic DNA extraction were germinated in vermiculite for 2 wk at 30° under standard greenhouse conditions. The second true leaf of three plants from each line was excised and put on ice. A total of 12 leaf samples were frozen with liquid nitrogen and lyophilized for 3 d. After the samples were freeze-dried, they were ground in a mortar and pestle with liquid nitrogen until a fine powder was produced. The powder was transferred to a 1.5-ml collection tube and DNA was isolated using the DNeasy Plant Mini Kit protocol (QIAGEN, USA). DNA concentrations were measured with the Nanodrop Spectrophotometer ND-1000 Version 3.3.0.

Plants for total RNA extraction were germinated in soil (Metro Mix 360 Growing Medium) on a hot-pad set at 30° under standard greenhouse conditions and grown for 17 d. Young true leaves from three plants of each line were immediately frozen in liquid nitrogen. To eliminate RNAses, all materials were cleaned with RNaseZap (Ambion, USA) before and during the extraction procedure. Tissues were ground in precooled mortars and pestles in liquid nitrogen. RNA extraction used RNeasy Plant Mini Kit following manufacturer’s instructions (QIAGEN, USA), including DNA digestion (RNase-Free DNase Set #79254; QIAGEN, USA). Potential DNA contamination was assessed by the polymerase chain reaction (PCR) following DNAse digestions. PCR reactions were: 12.5 µl of EconoTaq PLUS GREEN 2× Master Mix, 1 µL of 5 µM of each of *actin3* forward (5′- CCA AGG CGA ATA GAG AGA AAA-3′) and reverse (5′- GCA ACA TAC ATA GCG GGA GTG-3′) primers, 1 µl of RNA sample and 9.5 µL of ddH_2_O, for a total volume of 25 µl. The PCR program was 3 min at 94°, 30 sec at 94°, 30 sec at 60°, and 30 sec at 72° for 30 cycles and 5 min at 72° for final extension. Samples that produced an amplicon were redigested with DNAse following the manufacturer’s protocol (QIAGEN, USA). Concentrations of RNA in DNA-free samples were measured using the Nanodrop Spectrophotometer ND-1000 Version 3.3.0.

### Isolation of mitochondria for DNA and protein extraction

Mitochondrial isolations were performed for all lines by differential centrifugation through Percoll-density gradients. Fifteen plants were grown of each line for 45 d. For each line, three individual plants were used as one biological replicate, with a total of three biological replicates. A total of 50 g of green leaf tissue was used for mitochondrial isolations. Buffers from Millar *et al.* (2001) and extraction procedure from Lang *et al.* (2011) were used in this study with modifications. Buffers used were: homogenization buffer [400 mM mannitol, 1 mM EGTA, 25 mM MOPS-KOH, pH 7.8, 10 mM tricine, 8 mM cysteine (added the day of the extraction), 0.1% (w/v) BSA and 1% (w/v) PVP-40], washing buffer (400 mM mannitol, 1 mM EGTA, 10 mM MOPS-KOH, pH 7.2, and 0.1% BSA), and 5× gradient buffer [1.5 M sucrose, 50 mM MOPS-KOH, pH 7.2, and 0.5% (w/v) BSA]. Percoll density gradients were made in 1× gradient buffer in 30-ml Corex tubes (DuPont Instruments, USA). Centrifugation was performed using GSA and SS34 rotors in a Sorvall RC-5B refrigerated centrifuge at 4° (DuPont Instruments, USA).

The leaf tissue was chopped in a blender with 400 ml of homogenization buffer for 15 sec at the low setting. This was repeated three times. The processed tissue was filtered through eight sheets of cheesecloth and two sheets of Miracloth (Calbiochem, San Diego CA, USA) into a 1-liter beaker in ice. The filtrate was put in two 250-ml flasks and centrifuged in a GSA (Sorvall, USA) rotor at 1000 × *g* for 10 min to remove cellular debris. The supernatant was transferred to two new 250-ml flasks and was centrifuged at 3000 × *g* for 10 min to remove the nucleus from the homogenate. The resulting supernatant was transferred to two new 250-ml flasks and centrifuged at 17,000 × *g* for 20 min to pellet mitochondria. The pellet was resuspended with a soft-fine brush in the remaining homogenizing buffer. Corex tubes contained 10 ml of 80% and 33% Percoll in 1× gradient buffer. The 2 ml of resuspended mitochondrial pellet was transferred with a plastic Pasteur pipette to the Percoll gradient slowly. The corex tubes were put in the SS34 rotor and centrifuged at 18,000 × *g* for 60 min. A pale white band was extracted from the 33–80% interface with a plastic Pasteur pipette carefully. The mitochondrial extract was washed twice with 15 ml of washing buffer by centrifugation at 18,000 × *g* for 20 min each time. Pellets were stored at −80°.

### Sequencing and analysis

The mtDNA from inbred B and MSC 3, 12, and 16 were individually barcoded and one plate was sequenced at the UW Biotech Center using the Roche 454 FLX platform and protocols as recommended by the manufacturer (Roche, Branford, CT, USA). Reads were aligned to the mitochondrial genome of cucumber (Alverson *et al.* 2011) using the Integrative Genomics Viewer (IGV) version 2.3.32 (37) (Robinson *et al.* 2011). We surveyed mitochondrially encoded genes whose products are subunits in different mitochondrial complexes. In addition, mitochondrially encoded ribosomal proteins were evaluated for coverage. For the target genes, the numbers of reads were counted at the first, middle, and last nucleotide to obtain an average read coverage for the gene. Once the average coverage of the gene was established, it was divided by the total average read coverage of the whole mitochondrial genome alignment. Genes that were significantly different in read coverage were further evaluated for DNA and RNA amounts.

### Mitochondrial gene copy number and transcript levels measurements by using quantitative polymerase chain reaction

A total of 500 ng of DNA-free RNA from each sample was used to synthesize cDNA according to manufacturer’s recommendation with random primers (EasyScript cDNA Synthesis Kit, Lamda Biotech, USA). After reverse-transcription (RT), PCR reactions were used to verify that the cDNA synthesis was successful by amplifying to *gadph* gene. The samples were adjusted to an approximate concentration of 2 ng/µl. Quantitative PCR was performed using genomic DNA and cDNA with mitochondrial and nuclear gene-specific primers (Supporting Information, Table S4) on iCycler instrument (Bio-Rad Laboratories, USA) with Maxima SYBR Green/Fluorescein qPCR Master Mix (2×) (Thermo Scientific, USA). Reactions were in a volume of 25 µl with primer concentrations of 0.5 µM. Relative quantifications using the maximum curvature approach were performed using the iCycler IQ Optical System Software version 3.0a (Bio-Rad Laboratories, USA). MSC lines were compared to wild-type B after normalization with the nuclear *gadph* gene (Csa1M050240.4) as the reference because we detected no expression-level differences among B and the MSC lines (Table S3). The amplification protocol was 95° for 10 min, 40 cycles at 95° for 30 sec, 60° for 30 sec, 72° for 30 sec with data collection, and 55° for 15 sec. Verification of amplicon fidelity was performed through melt curve analysis. All primers for mitochondrial genes were designed from the Calypso Mitochondrial Genome (HQ860792; Alverson *et al.* 2011) and nuclear genes from the Cucurbit Genomics Database (*actin3*, Csa3M806800.1; *gadph*, Csa1M050240.4; and *ubqc*, Csa1M000740.1; www.icugi.org) (Table S4).

Calculations for fold change were performed using the 2^−ΔΔCt^ method described in detail by Livak and Schmittgen (2001). Three technical replicates were performed in each of the samples to calculate for statistical significance using a Student *t*-test comparing each MSC line to wild-type B. Gene expression was considered significantly different when the *P* value was less than 0.05.

### Statistical analysis

Pairwise Student *t*-test comparisons between an MSC line with wild-type inbred B for fold changes in copy number and transcript levels were performed for each gene evaluated in this study. The ratio between the average coverage of a specific gene over the overall read coverage of a given mitochondrial line (Table 1), as well as the ratio between a protein of interest over PORIN (Figure 4, B and C), were log_10_-transformed to perform the pairwise *t*-test comparison between an MSC and wild-type inbred B. *P* values less than 0.05 were considered significantly different.

**Table 1:** Fold-change differences for average read coverage of mitochondrial genes

Gene	Position in Reference	Function	Inbred B ARC^a^	MSC3 ARC^a^	MSC12 ARC^a^	MSC16 ARC^a^
Mt DNA			24.6	64.6	7.9	19.2
*nad9*	1,406,569-1,407,141	Complex I	1.41	1.07	**2.42***^b^*^,^*^d^*	1.70
*nad7*	696,357-706,848		1.45	1.22	0.84	1.89
*nad6*	767,315-767,938		1.09	1.38	1.39	1.18
*nad3*	711,309-711,665		1.33	1.51	1.47	1.25
*nad5 ex4*	417,477-419,145		1.11	**0.28***^b^*^,^*^c^*	0.76	0.88
*sdh3*	793,582-793,899	Complex II	1.00	1.27	1.27	0.88
*cob*	377,338-378,504	Complex III	0.80	0.98	0.51	0.83
*cox1*	1-2,592	Complex IV	1.33	1.31	1.01	1.63
*cox2*	1,428,065-1,428,847		1.37	1.32	1.22	1.18
*ccmFc*	4,099- 6,394		0.93	**1.27***^b^*^,^*^d^*	1.10	**1.54***^b^*^,^*^d^*
*ccmB*	710,286-710,906		1.72	1.47	1.05	1.79
*atp1*	1,402,356-1,403,879	Complex V	1.37	1.35	1.64	1.54
*atp4*	419,518-420,114		0.57	**0.12***^b^*^,^*^c^*	0.63	0.83
*atp8*	1,420,574-1,421,053		1.00	1.06	**1.69***^b^*^,^*^d^*	1.45
*rpl2*	1,023,028-1,024,835	Ribosome LU	1.35	1.40	1.81	1.56
*rpl5*	1,189,999-1,190,589		1.34	0.86	1.27	1.44
*rrnL*	833,044-836,629		2.95	3.15	3.12	3.59
*rrn5*	917,679-917,792	Ribosome SU	1.68	1.96	**1.18***^b^*^,^*^c^*	1.56
*rrnS*	917,977-919,801		1.58	2.65	4.51	3.75
*rps7*	261,085-261,531		1.08	0.68	**0.13***^b^*^,^*^c^*	**0.14***^b^*^,^*^c^*
*rps13*	1,288,876-1,289,226		1.18	1.22	1.35	0.94
*rps10*	1,554,211-1,555,628		0.83	**1.26***^b^*^,^*^d^*	1.56	**1.80***^b^*^,^*^d^*
*rps3*	736,620-742,986		1.46	1.56	1.35	1.27

Fold-change differences for average read coverage (ARC) of mitochondrial genes from wild-type inbred B and mosaic (MSC) lines 3, 12, and 16 normalized to the ARC across the entire mitochondrial DNA of each line.

a ARC, average read coverage. This refers to the overall read coverage in the mtDNA.

b Bold text indicates significant difference at α = 0.05.

c Under-represented region relative to wild-type inbred B.

d Over-represented region relative to wild-type inbred B.

### Proteomic analysis

#### Enzymatic “in-liquid” digestion:

Mitochondrial extracts were methanol:chloroform–extracted for their protein content based on work by Wessel and Flügge (1984). “In liquid” digestion and mass spectrometric analysis was performed at the Mass Spectrometry Facility (Biotechnology Center, University of Wisconsin-Madison). A total of 50 µg of extracted mitochondrial proteins was resolubilized and denatured in 30 µl of 8 M urea/50 mM NH_4_HCO_3_ (pH 8.5)/1 mM Tris-HCl (pH 7.5) for 10 min, then diluted for reduction with 5 µl of 25 mM DTT, 10 µl methanol, and 75 µL 25 mM NH_4_HCO_3_ (pH 8.5). Samples were reduced for 15 min at 55° and then cooled, followed by an alkylating step with 6 µl of 55 mM IAA for 15 min at room temperature. Reaction was terminated by adding 12 µl of 25 mM DTT and digestion was commenced by adding 8 µL Trypsin [100 ng/μl *Trypsin Gold* (PROMEGA Corp.) in 25 mM NH_4_HCO_3_], 4 µl LysC [100 ng/μl Lysyl endopeptidase (Wako Pure Chemical Industries, Ltd.) in 25 mM NH_4_HCO_3_], and 50 µl of 25 mM NH_4_HCO_3_ (pH 8.5). Digestion was conducted for 2 hr at 42° and then additional 4 µl of trypsin and 2 µl of LysC solutions were added and digestion proceeded overnight at 37°. Reaction was terminated by acidification with 2.5% trifluoroacetic acid (TFA) to 0.3% final.

#### NanoLC-MS/MS:

Digests were cleaned using OMIX C18 SPE cartridges (Agilent, Palo Alto, CA) using the manufacturer’s protocol and eluted in 25 µl of 60/40/0.1% ACN/H_2_O/TFA, dried to completion in the speed-vac, and finally reconstituted in 100 µL of 0.1% formic acid. Peptides were analyzed by nanoLC-MS/MS using the Agilent 1100 nanoflow system (Agilent, Palo Alto, CA) connected to a hybrid linear ion trap-orbitrap mass spectrometer (LTQ-Orbitrap XL; Thermo Fisher Scientific, Bremen, Germany) equipped with a nanoelectrospray ion source. Chromatography of peptides prior to mass spectral analysis was accomplished using capillary emitter column (in-house packed with MAGIC C18, 3 µM, 150 × 0.075 mm; Michrom Bioresources, Inc.), onto which 4 µl (∼2 µg) of extracted peptides were automatically loaded. NanoHPLC system delivered solvents A [0.1% (v/v) formic acid in water] and B [95% (v/v) acetonitrile, 0.1% (v/v) formic acid] at either 0.5 µl/min to load sample or 0.20 µl/min to elute peptides directly into the nano-electrospray over a 145-min 0% (v/v) B to 40% (v/v) B followed by 20 min 40% (v/v) B to 60% (v/v) B gradient. As peptides eluted from the HPLC-column/electrospray source survey, MS scans were acquired in the Orbitrap with a resolution of 100,000 and up to 5 of the most intense peptides per scan were fragmented and detected in the ion trap over the 300 to 2000 m/z; redundancy was limited by dynamic exclusion. Raw MS/MS data were searched against *Cucumis sativus* amino acid sequence database (42,111 protein entries) using in-house *Mascot* search engine 2.2.07 (Matrix Science) with variable Methionine oxidation with asparagine and glutamine deamidation. Peptide mass tolerance was set at 15 ppm and fragment mass was set at 0.6 Da. Protein annotations, significance of identification, and spectral-based quantification were performed with help of Scaffold software (version 3.6.3; Proteome Software Inc., Portland, OR). Protein identifications were accepted if they could be established at more than 95.0% probability within 0.9% false discovery rate and contained at least two identified peptides. Protein probabilities were assigned by the Protein Prophet algorithm (Nesvizhskii *et al.* 2003). Proteins that contained similar peptides and could not be differentiated based on MS/MS analysis alone were grouped to satisfy the principles of parsimony. Statistical differences were based on pairwise comparisons using a *t*-test between an MSC mutant and wild-type B after the quantity of peptides detected was normalized by weighted spectra.

### Western blot analysis

Twenty 20-µl of total leaf tissue protein extract in 1× SDS sample buffer [50 mM Tris-HCl pH 6.8, 2% (w/v) SDS, 10% (v/v) glycerol, 5% (v/v) β-mercaptoethanol, 12.5 mM EDTA, and 0.02% bromophenol blue] were run in a 12.5% polyacrylamide gel for 50 min at 200 V to separate proteins. The Bullseye Protein Ladder (BEPAR) was used to determine the molecular weights of the proteins (MIDSCI). The SDS-PAGE running buffer was 1× of Tris/Glycine/SDS (Bio-Rad #161-0772). The transfer membrane (Immobilon-FL PVDF; Millipore) was incubated in 100% methanol for 5 min, followed by a wash with transfer buffer [1× Tris/Glycine buffer (Bio-Rad #161-0734) and 2% v/v methanol] for 5 min. The membrane and gel were put between two pieces of filter paper (Grade GB003; Whatman) and introduced to the transfer apparatus (Criterion Blotter; Bio-Rad) for 30 min at 100 V.

Once the transfer occurred, the membrane was blocked with 5% milk (for PORIN and COX2) or 5% BSA (for ATP4) in 1× PBS buffer (137 mM NaCl, 2.7 mM KCl, 10 mM Na_2_HPO_4_, and 1.8 mM KH_2_PO_4_) for 1 hr. Subsequently, incubation with the primary antibodies (1:1000) was performed for 1 hr. Afterward, the membrane was washed one time with 1× PBS for 10 min, followed by two washes with 1× PBS 0.1% (v/v) Triton X-100 for 10 min each, ending with one wash with 1× PBS for 10 min. Secondary antibody (anti-mouse for PORIN, anti-rabbit for ATP4 and COX2; LI-COR) incubation was performed for 1 hr at 1:10,000 dilution. Reactive bands were measured using Odyssey infrared imaging system (LI-COR).

## Results

### Sequencing revealed under-represented regions in the mtDNA of MSC lines

We used the Roche 454 platform to sequence the mtDNAs of wild-type inbred B and MSC 3, 12, and 16 (Genbank Sequence Read Archive SRP051771), each independently derived from inbred B and showing distinct paternally transmitted mosaic phenotypes (Figure 1) (Bartoszewski *et al.* 2007). The reads were aligned to the mtDNA reference sequence of "Calypso" (Alverson *et al.* 2011). Although we acknowledge that the mtDNA of inbred B is not necessarily identical to "Calypso," the sequences of known mitochondrial genes agreed between both lines and were the focus of this research. The average read coverages (ARC) of specific genes in the mtDNA of each MSC line were adjusted to the ARC across the entire mtDNA to reveal those with significantly reduced or increased copy number relative to wild-type B (Table 1). Previous work showed that MSC16 has a large deletion (JLV5-Del) in its mitochondrial genome (Lilly *et al.* 2001), and our sequencing confirmed this deletion from 347,555 to 362,667 bp (15.1 kb) of the reference mtDNA of "Calypso" (Alverson *et al.* 2011) (Table S1). We observed different under-represented regions in the three MSC lines as compared to inbred B (Table S1). MSC3 had 131.6 kb (7.8%), MSC12 had 242.5 kb (14.4%), and MSC16 had 258.5 kb (15.3%) either missing or under-represented relative to the "Calypso" reference (Table S1), which could interfere with the overall function of the mitochondria.

ARC of 23 mitochondrial genes (adjusted to the ARC across the entire mtDNA) were determined and 78%, 83%, and 87% of these genes were not statistically different from the ARC of wild-type B for MSC3, MSC12, and MSC16, respectively (Table 1). However MSC3 had significant (*P* < 0.05) under-representation of the polycistronic region carrying *nad5ex4-atp4-nad5ex5*, which codes for exons 4 and 5 of NADH dehydrogenase subunit 5 and ATPase subunit 4 (Table 1). In addition, MSC3 had an over-representation of the cytochrome c biogenesis FC (*ccmFc*), 18S ribosomal RNA (*rrnS*), and ribosomal protein S10 (*rps10*) (Table 1) (*P* < 0.05). MSC12 and MSC16 have an under-representation of ribosomal protein S7 (*rps7*) as compared to wild-type B (Table 1) (*P* < 0.05). In addition, MSC12 has under-representation of the 5S ribosomal RNA (*rrn5*) (Table 1) (*P* < 0.05), as well as more coverage depth of NADH dehydrogenase subunit 9 (*nad9*) and ATPse subunit 8 (*atp8*). MSC16 had over-representation of *ccmFc* and *rps10* (Table 1) (*P* < 0.05).

### Quantitative PCR confirmed copy number differences among MSC lines and wild-type B

**Figure 2:**
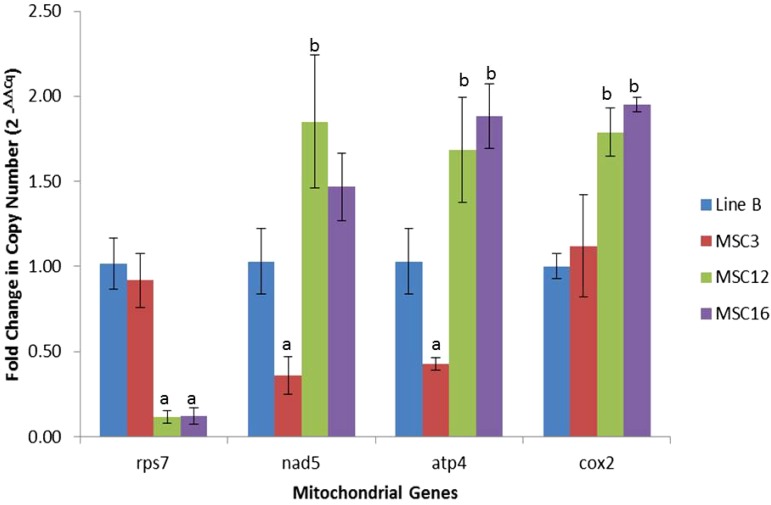
Fold-change differences with standard errors for copy number of mitochondrial genes among wild-type line B and MSC lines. Normalization was performed using the nuclear gene *gadph* as compared to wild-type B as described by Livak and Schmittgen (2001). Significant difference between an MSC mutant for a given mitochondrial gene as compared to wild-type B was established at α = 0.05 using pairwise *t*-test. Lower and higher copy number comparison between an MSC mutant and wild-type line B are shown as “a” and “b,” respectively.

We focused on mitochondrial genes that appeared in lower copy number in MSC3, MSC12, and MSC16 relative to wild-type B (Table 1 and Table S1). The polycistronic region *nad5ex4-atp4-nad5ex5* in MSC3 and *rps7* in MSC12 and MSC16 were hypothesized to be likely candidates for their respective MSC phenotypes and their relative amounts were assessed using quantitative (q) PCR (Figure 2 and Table S1). Normalization was performed using the nuclear gene *gadph*, because it has been previously validated as an appropriate reference gene for leaf tissues (Hruz *et al.* 2011), and we did not detect copy number differences among the MSC lines and wild-type B (Table S2).

Copy numbers of mitochondrial genes were similar for MSC3 and wild-type B except for significantly (*P* < 0.05) fewer copies of *nad5ex4* and *atp4* (0.36 ± 0.11X and 0.43 ± 0.04X, respectively) and higher copy number of *cob* (1.29 ± 0.05X, apocytochrome b) (Figure 2 and Table S2). *Cob* did not show a difference in sequence coverage depth as compared to the wild-type, but did so using qPCR (Table S2). As expected from the next-generation sequencing results, *rps7* had significantly lower copy number at 0.12 ± 0.04X for both MSC12 and 16 relative to B (Figure 2 and Table S2). Interestingly, MSC12 and MSC16 had an average of two-fold increased copy number for some mitochondrial genes (up to 2.9-fold) relative to wild-type B (Figure 2 and Table S2).

### Mitochondrial genes in under-represented regions have fewer transcripts compared to wild-type B

**Figure 3:**
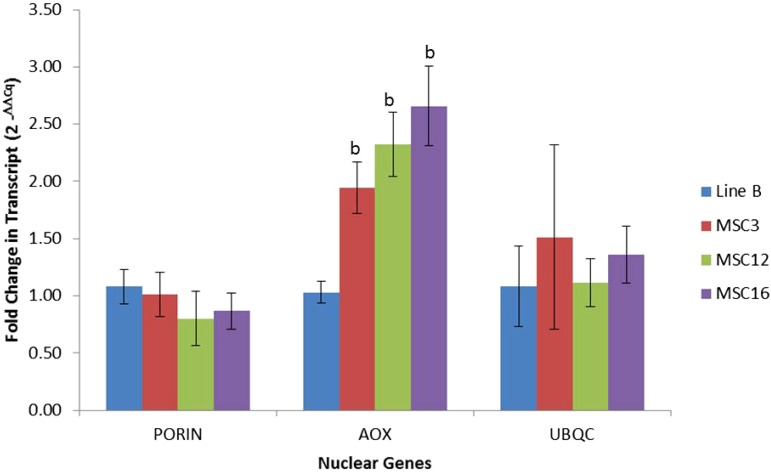
Fold-change difference in nuclear-gene transcript levels between wild-type line B and the MSC lines. *Porin* and alternative oxidase (*aox*) are nuclear-encoded mitochondrially targeted proteins. Ubiquitin C (*ubqc*) is nuclear-encoded gene that functions in the cytoplasm. Normalization was performed using the nuclear gene *gadph* as compared to wild-type B as described by Livak and Schmittgen (2001). Significant difference between an MSC mutant for a given nuclear gene as compared to wild-type B was established at α = 0.05 using pairwise *t*-test. Lower and higher copy number comparison between an MSC mutant and wild-type line B are shown as “a” and “b,” respectively.

**Figure 4:**
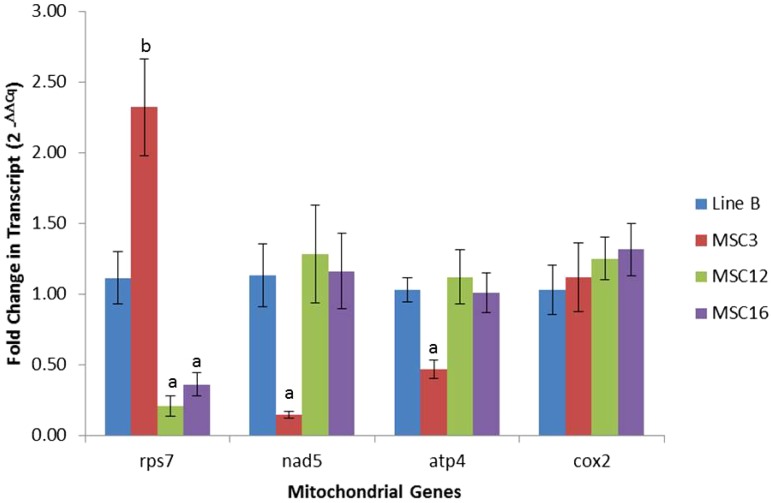
Fold-change differences with SEs for mitochondrial genes transcript levels of mitochondrial genes among wild-type line B and MSC lines. Normalization was performed using the nuclear gene *gadph* as compared to wild-type B as described by Livak and Schmittgen (2001). Significant difference between an MSC mutant for a given mitochondrial gene as compared to wild-type B was established at α = 0.05 using pairwise *t*-test. Lower and higher copy number comparison between an MSC mutant and wild-type line B are shown as “a” and “b,” respectively.

No significant differences were observed for the nuclear transcripts *porin* and *ubqc* between wild-type B and the MSC lines (Figure 3). As expected, expression of alternative oxidase (*aox*) was significantly higher in the MSC lines relative to B; AOX is nuclear-encoded protein and shows upregulation in stressed plants and mitochondrial mutants (Karpova *et al.* 2002, Juszczuk *et al.* 2007, Polidoros *et al.* 2009). MSC3 showed lower transcript abundance for *nad5ex4* (0.14 ± 0.02X) and *atp4* (0.47 ± 0.07X) as compared to wild-type B (Figure 4). MSC12 and MSC16 had eight-fold fewer transcripts for *rps7* as compared to wild-type B (Figure 4 and Table S3). In addition, MSC12 had lower (0.5 ± 0.12X) expression levels for *rrnS* (18S rRNA) (Table S3). Surprisingly, all three MSC lines had significantly higher (*P* < 0.05) expression levels for *nad9*, *nad6*, *nad3*, *sdh3*, *ccmFc*, *cob*, *ccmB*, *cox1*, *atp1*, *rps3*, *rpl2*, *rps13*, *rpl5*, and *rps10* as compared to wild-type B (Table S3). These results suggest that the genetic basis of the mosaic phenotype of MSC3 may be lower amounts of the *NAD5* and *ATP4* proteins, potentially resulting in unstable complexes for NADH dehydrogenase (Complex I) and ATP synthase (Complex V). In the case of MSC12 and MSC16, both may have compromised function of the small subunit in the mitochondrial ribosome due to lower abundance of the *RPS7* protein.

### Liquid chromatography-mass spectrometry reveals no significant differences in protein quantity for mitochondrially encoded proteins but does for nuclear-encoded mitochondrially targeted proteins

Mitochondrial protein extracts from wild-type B, MSC3, MSC12, and MSC16 were evaluated by LC-MS to estimate their relative amounts. A total of 483 proteins were confidently identified, including mitochondrially encoded *ATP1*, *ATP4*, *ATP8*, *NAD7*, and *NAD9* (Table 2). Amounts of most of these proteins were not statistically different except *NAD7*, which was significantly (*P* < 0.05) lower in MSC3 and MSC12 as compared to wild-type B. *FTSH4*, a nuclear-encoded ATP-dependent mitochondrial protease, had significantly (*P* < 0.05) higher amounts in MSC16 (Table 2).

**Table 2:** Mass spectrometry estimates of the normalized weighted spectra

Protein	Line B	MSC3	MSC12	MSC16
ATP1	45.3 ± 13.3	36.1 ± 8.2	32.9 ± 10.9	36.2 ± 8.7
ATP4	4.4 ± 1.6	5.0 ± 1.2	4.1 ± 1.4	5.8 ± 1.5
ATP8	3.9 ± 1.3	4.6 ± 1.7	4.8 ± 1.2	4.4 ± 0.8
NAD7	2.6 ± 0.1	**1.9 ± 0.0***^a^*	**1.5 ± 0.7***^a^*	1.7 ± 0.7
NAD9	2.1 ± 0.6	4.2 ± 1.3	3.4 ± 1.3	1.4 ± 0.8
FTSH4	2.1 ± 0.4	4.0 ± 2.6	3.0 ± 1.4	**4.4 ± 0.3***^a^*

Mass spectrometry estimates of the normalized weighted spectra (± SD) of mitochondrial-encoded proteins from wild-type B, MSC3, MSC12, and MSC16.

a Estimates for MSC lines were significantly different from B at α = 0.05 using *t*-tests with two samples and unequal variance.

### Western blots reveal no consistent differences for quantities of mitochondrial proteins in the MSC lines as compared to wild-type B

**Figure 5:**
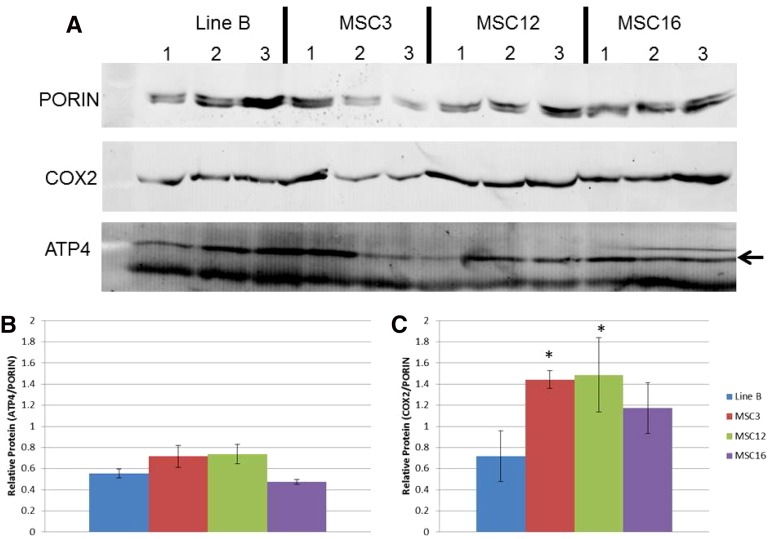
Western blot analyses (A) of amounts of COX2 and ATP4 (arrow) proteins in three replicated samples of protein from plants of wild-type B and MSC3, MSC12, and MSC16. Relative amounts of ATP4 and COX2 (B and C) were estimated after normalization to amounts of nuclear-encoded mitochondrially targeted PORIN. Statistical analyses were performed using log_10_ of the ratio, and the asterisk indicates significantly greater amounts of COX2 protein in the MSC3 and MSC12 as compared to B at *P* < 0.05.

Western blots were performed using whole leaf protein extracted from wild-type B, MSC3, MSC12, and MSC16 to evaluate the quantity of *ATP4* as the potential genetic basis of MSC3, and using COX2 as a representative mitochondrially encoded protein for assessing potentially compromised mitochondrial translation in MSC12 and MSC16 (Figure 5A). The results revealed that amounts of the *ATP4* protein in the MSC lines were similar to wild-type B (Figure 5B). For *COX2*, MSC12 had a significantly (*P* < 0.05) increased amount of the *COX2* protein as compared to wild-type B, and MSC16 was not significantly different (Figure 5C).

## Discussion

Our study focused on potential genetic bases of three independently produced mitochondrial mutants of cucumber: MSC3, MSC12, and MSC16. These mutants were recovered after regeneration of plants from different cell-culture experiments established using the highly inbred wild-type Line B (Figure 1) (Bartoszewski *et al.* 2007). Because cucumber, unlike most eukaryotes, shows paternal transmission of the mitochondria, mitochondrially associated phenotypes can be established by reciprocal crossing (Havey 1997; Havey *et al.* 1998). It was known that these three MSC lines possess different mtDNA conformations (Bartoszewski *et al.* 2007), and that MSC16 has a major deletion in its mtDNA (Lilly *et al.* 2001), although no coding regions were found in this deleted region. Our sequencing results confirmed this deletion in the mtDNA of MSC16, as well as in MSC12 (Table S1). Sequencing of the mtDNAs of MSC3, MSC12, and MSC16 revealed different missing or under-represented regions relative to B, indicating that independently derived MSC phenotypes do not trace back to the same sublimon in B. Because numerous MSC lines have been identified from independent cell-culture experiments (Malepszy *et al.* 1996; Ładyżyński *et al.* 2002), cucumber may provide a system to select for rearrangements in the mtDNA that affect mitochondrial gene expression. We hypothesized that the cause of the MSC phenotypes could be due to either deleted or lower copy regions in the mtDNA relative to wild-type plants.

### Reduced transcript levels NADH dehydrogenase subunit 5 (*nad5*) and ATP synthase subunit 4 (*atp4*) associated with the MSC3 phenotype

The MSC3 phenotype is associated with the relatively lower copy number and transcript abundance of *nad5* and *atp4* (Table S2 and Table S3), both part of a polycistronic region. These genes are parts of complexes I and V, respectively, of the electron transport chain in the mitochondrion. Because the effectiveness of energy production relies on the coupling of these complexes in the inner membrane of the mitochondrion, missing subunits could result in leakage of electrons as they are being transported through the complexes. MSC3 would be similar to NCS mutants of maize as an example of under-representation of mitochondrial-encoded genes in the electron transport chain (Lauer *et al.* 1990, Newton *et al.* 1990, Karpova and Newton 1999). However, mass spectrometry and Western blots revealed no significant differences for amounts of the ATP4 protein in the mitochondria of MSC3 and wild-type inbred B (Figure 5, A and B). Subunit 5 of NADH dehydrogenase (*NAD5*) was not tested because of the lack of the *NAD5* antibody. Future studies using respiration assays may provide a clearer assessment of the efficiency of respiration in this mitochondrial mutant.

### Ribosomal protein S7 (*rps7*) in the mitochondrial mutants MSC12 and MSC16

We observed that MSC12 and MSC16 had lower copy number and transcript amounts of the ribosomal protein S7 (*rps7*) (Table 1, Figure 2, Figure 4, Table S2, and Table S3). In *Escherichia coli*, the *rpsG* (homolog of eukaryotic *rps7*) gene encodes for a first-hierarchy protein in the small subunit of the ribosome, binds to the 3′-end of the bacterial 16S rRNA, and interacts with second-hierarchy ribosomal proteins S9, S13, and S19 (reviewed by Shajani *et al.* 2011). It has been demonstrated that the binding of *rpsG* to the bacterial 16S rRNA makes the head of the small subunit of the ribosome to recognize mRNA and initiate translation (Spencer *et al*. 1984; Chapel *et al.* 1987; Robert and Brakier-Gingras 2001; Bunner *et al.* 2010). Similarly, *rps7* in the chloroplast of *Chlamydomonas* is associated with translation initiation (Fargo *et al.* 2001).

We evaluated copy numbers and transcript amounts of the mitochondrial genes *rps3*, *rps10*, and *rps13* because they are part of the 3′-domain in the prokaryotic small subunit of the ribosome (Bunner *et al.* 2010). These genes had double the copy numbers and transcript levels as compared to wild-type B (Table S2 and Table S3, respectively). In *Arabidopsis*, silencing of the nuclear-encoded mitochondrially targeted *rps10* resulted in a doubling of mtDNA as well as increased transcription across all mitochondrial genes (Kwasniak *et al.* 2013). Our data also show an unexpected increase in relative copy numbers as well as transcript levels, but not across all genes. Out of the 23 genes evaluated in this study, only *nad7*, *rrn5*, *ccmB* (in MSC16), and *atp4* (in MSC12) had statistically the same copy numbers as compared to wild-type B. Interestingly, not all transcripts were increased but genes encoding proteins in Complex I (*nad9*, *nad6*, *nad3*, *nad5*, and *nad7*) showed on average two-fold more transcripts as compared to wild-type B (Table S2 and Table S3).

It has been hypothesized that the basis of the MSC16 phenotype is due to low quantities of complex I proteins resulting in higher levels of reactive oxygen species (Juszczuk and Rychter 2009). Our results show that genes of complex I are expressed at relatively high levels, suggesting that mitochondrial genes in this complex might be transcribed normally. However, the small quantities of complex I proteins observed by Juszczuk and Rychter (2009) could be due to defective translational machinery, such as a compromised small ribosomal subunit, rather than a defective or under-represented protein in this complex as may be the case for MSC3.

### Cucumber as a model to produce mitochondrial transcript “knock-downs”

Mutations or deletions in the mtDNAs of model plants such as *Arabidopsis*, tobacco, and maize have been described and revealed insights about mitochondrial energy production and nuclear interactions (Cherit *et al.* 1992; Kanazawa *et al.* 1994; Karpova and Newton 1999; Kuzmin *et al.* 2005; Shedge *et al.* 2007). One disadvantage with these plants is that their organelles are maternally transmitted, not allowing for separation of any putative chloroplast or mitochondrial effects. It is also likely that efficient production of mitochondrial mutants will be difficult due to the relatively large numbers of mtDNAs in each mitochondrion and mitochondria per cell, as well as potential lethality associated with the loss of a mitochondrial gene. A proposed alternative approach would be to produce mitochondrial mutants by knocking out nuclear genes that encode mitochondrially targeted proteins (Colas Des Francs-Small and Small 2014).

Cucumber is a useful model plant for mitochondrial genetics because differential transmission of the organelles allows for separation of mitochondrial and chloroplast effects by simple reciprocal crossing (Havey 1997; Havey *et al.* 1998, 2002), a relatively large mtDNA that shows structural diversity among closely related plants (Havey 1997; Alverson *et al.* 2011), the likely transmission of relatively few mitochondria through the male gametophyte to progenies (Abreu *et al.* 1982; Lilly *et al.* 2001), and the appearance of phenotypically distinct, paternally transmitted MSC phenotypes after passage of wild-type cucumber through cell cultures (Malepszy *et al.* 1996; Ładyżyński *et al.* 2002; Bartoszewski *et al.* 2007). Because independently derived MSC lines possess different mtDNA rearrangements relative to inbred B (Bartoszewski *et al.* 2004b), they have been proposed as a system to produce mitochondrial mutants (Bartoszewski *et al.* 2007). In this study, we evaluated for differences in the genome, transcriptome, and proteome of three MSC lines, each one independently derived from different cell-culture experiments established using wild-type inbred B (Figure 1), to study potential genetic bases of the MSC phenotypes.

Sequencing revealed and qPCR confirmed that specific regions are significantly under-represented in the mtDNAs of the MSC lines relative to wild-type progenitor (Table 1 and Table S2). Because complete absence of a mitochondrial gene would likely be lethal (Mackenzie *et al.* 1994; Colas Des Francs-Small and Small 2014), significant under-representation of coding regions may disturb mitochondrial function to produce the mosaic phenotypes. In addition to under-represented regions, we observed that other mitochondrial regions were significantly over-represented in the MSC lines relative to B (Table 1 and Table S2). This observation is in agreement with the models proposed by Albert *et al.* (1998), in which deletions of specific mitochondrial regions should be associated with duplications of other regions.

Genes carried on these under-represented regions produced significantly fewer transcripts relative to both inbred B and mitochondrial genes that were not under-represented in the MSC lines (Table S3). These results agree with the significantly fewer transcripts associated with specific genes in the NCS mutants of maize (Newton *et al.* 1990; Hunt and Newton 1991); however, our results do not agree with observations that no relationship exists between DNA copy numbers and transcript levels in the mitochondria of different bean (*Phaseolus vulgaris*) cytoplasms (Woloszynska *et al.* 2006).

Based on differences for DNA copy numbers and transcript amounts, we hypothesized that the basis of MSC3 phenotype may be dysfunctional mitochondrial complex I and/or complex V due to lower amounts of the *NAD5* and *ATP4* proteins, respectively. Western blots demonstrated that amounts of the *ATP4* protein were the same in MSC3 as wild-type B (Figure 5B), even though DNA copy number and transcript amounts were significantly reduced (Figure 2, Figure 4). For MSC12 and MSC16, reduced transcription of *rps7* may compromise the assembly of the small subunit of the mitochondrial ribosome, causing reduced ribosome stability and/or inefficient protein production. However, mass spectrometry revealed no significant differences for mitochondrially encoded proteins between these MSC lines and B (Table 2). Western analysis of *COX2*, a mitochondrially encoded protein with double copy number (Figure 2) but similar transcript amounts (Figure 4) in the MSC lines and B, showed no significantly different amounts of this protein in MSC16, but was significantly higher in MSC12 as compared to wild-type B (Figure 5C). This result is not consistent with reports of less mitochondrially encoded proteins in the NCS3 and NCS4 mutants of maize, which lack an rps3/rpl6 coding region (Hunt and Newton 1991; Newton 1995). However, it is possible that *rps7* may regulate the translation initiation of specific gene(s). In *Chlamydomonas*, chloroplast *rps7* has been proposed as a translation initiation factor for *rps12*, *rbcL*, *atpB*, and *psbA*, based on crosslinking experiments in the 5′-UTR regions of these genes (Fargo *et al.* 2001). Although Fargo *et al.* (2001) did not test the protein production of these genes in their 5′-UTR *rps7* mutants, they suggest that free *rps7* might be a “docking” protein for these transcripts to the small subunit of the ribosome to initiate their translation.

In conclusion, our research demonstrates that independently produced MSC lines do not trace back to the same sublimon in inbred B, and that different regions of the mtDNA are significantly under-represented in MSC3, MSC12, and MSC16 relative to B. We observed significant agreement between copy number and transcript amounts for genes carried on these under-represented regions. Our results are also consistent with those of other researchers who reported poor correlations between amounts of mitochondrial transcripts and proteins (Kwasniak *et al.* 2013), possibly due to post-transcriptional differences such as translation efficiency or protein longevity (Giegé *et al.* 2000). Nevertheless, cucumber can be used to produce knock-downs of mitochondrial transcripts (Figure 4, Table S3). Mitochondrial DNA from independently produced MSC lines (Ładyżyński *et al.* 2002; Bartoszewski *et al.* 2007) can be sequenced to reveal under-represented genomic regions and reduced transcript levels for genes carried on these regions. These transcriptional “knock-downs” should be useful to study the dynamic nature of the plant mitochondrion and its interaction with a highly homozygous nucleus.

## Supplementary Material

Supporting Information
