# Supplementary material for: The Mosaic Mutants of Cucumber: A Method to Produce Knock-Downs of Mitochondrial Transcripts
Source: G3 (Bethesda). 2015 Apr 14;5(6):1211–21. doi: 10.1534/g3.115.017053 (PMC4478549; doi:10.1534/g3.115.017053)
Supplement: Supporting Information [file supp_5_6_1211__index.html]

The Mosaic Mutants of Cucumber: A Method to Produce Knock-Downs of Mitochondrial Transcripts — Supporting Information 

# The Mosaic Mutants of Cucumber: A Method to Produce Knock-Downs of Mitochondrial Transcripts

## Supporting Information for Del Valle-Echevarria *et al.*, 2015

**Files in this Data Supplement:**

- Supporting Information - Tables S1-S4 (PDF, 212 KB)
- Table S1 - Low-coverage regions in the mt DNA of the mitochondrial mutant MSC3, 12, and 16 as compared to the progenitor, wild-type inbred B. (PDF, 144 KB)
- Table S2 - Fold change of copy number of mitochondrial and nuclear genes in wild-type inbred B and mitochondrial mutants MSC3, 12 and 16. (PDF, 151 KB)
- Table S3 - Fold change in transcript levels of mitochondrial and nuclear genes from wild-type inbred B and mitochondrial mutants MSC3, 12 and 16. (PDF, 151 KB)
- Table S4 - Primer sequences that were used for quantitative-PCR. (PDF, 144 KB)
