# Supplementary material for: The Mosaic Mutants of Cucumber: A Method to Produce Knock-Downs of Mitochondrial Transcripts
Source: G3 (Bethesda). 2015 Apr 14;5(6):1211–21. doi: 10.1534/g3.115.017053 (PMC4478549; doi:10.1534/g3.115.017053)
Supplement: Supporting Information [file supp_g3.115.017053_017053SI.pdf]

G3 · Genes | Genomes | Genetics

Title: **The mosaic (MSC) mutants of cucumber: a method to produce knock-downs of mitochondrial transcripts**

Angel R. Del Valle-Echevarria[*], Agnieszka Kiełkowska[§], Grzegorz Bartoszewski[†], and Michael J. Havey[*,‡]

[*]Department of Horticulture and [‡]USDA Agricultural Research Service, University of Wisconsin, Madison, Wisconsin, USA

[§]Faculty of Horticulture, Agricultural University of Krakow, Al. 29 Listopada 54, 31-425 Krakow, Poland

[†]Department of Plant Genetics, Breeding and Biotechnology, Faculty of Horticulture, Biotechnology and Landscape Architecture, Warsaw University of Life Sciences, ul. Nowoursynowska 159, 02-776 Warsaw, Poland

Sequence reads have been deposited into Genbank Sequence Read Archive SRP051771 (release date June 1, 2015). IRB number for recombinant DNA research at the University of Wisconsin is SC12-008R.

Corresponding author:
Dr. Michael J. Havey
USDA-ARS
Department of Horticulture
1575 Linden Drive
University of Wisconsin
Madison, WI 53706 USA
Phone: 608-262-1830
Fax: 608-262-4743
Email: mjhavey@wisc.edu

DOI: 10.1534/g3.115.017053

Table S1 Low-coverage regions in the mt DNA of the mitochondrial mutant MSC3, 12, and 16 as compared to the progenitor, wild-type inbred B.

Mutant Line	Region (bp) Start	End	Size (bp)	Chromosome*	Annotation
MSC3	392,242	441,105	48,863	MC	*nad5ex4* (NADH dehydrogenase subunit 5 C-terminus); trnW-CCA (tRNA-Trp); *atp4* (ATPase subunit 4) and chloroplast-like DNA
	585,075	636,619	51,544	MC	
	1	31,162	31,161	83.8 kb	
		Tota	131,568	7.8%	
MSC12	149,606	161,705	12,099	MC	
	195,676	267,406	71,730	MC	*rps7* (ribosomal protein S7)
	347,550	362,669	15,119	MC	JLV5-Del (Lilly *et al.* 2001) was identified in MSC16)
	567,690	568,518	828	MC	
	572,554	622,942	50,388	MC	
	911,926	917,574	5,648	MC	
	22,564	25,406	2,842	44.8 kb	
	1	83,817	83,816	83.8 kb	
		Total	242,470	14.4%	
MSC16	149,606	161,706	12,100	MC	
	195,676	283,618	87,942	MC	*rps7* (ribosomal protein S7)
	347,550	362,669	15,119	MC	JLV5-Del (Lilly *et al.* 2001)
	567,711	568,451	740	MC	
	572,554	622,942	50,388	MC	
	911,926	917,574	5,648	MC	
	22,564	25,270	2,706	44.8 kb	
	1	83,817	83,816	83.8 kb	
		Total	258,459	15.3%	

MC = Master chromosome of 1.6 megabases (Mb).

44.8 kb = Extra-chromosome of 44.8 kb

83.8 kb = Extra-chromosome of 83.8 kb

*All locations are based on the mitochondrial sequence of Alverson *et al.* (2011).

Table S2 Fold change of copy number of mitochondrial and nuclear genes in wild-type inbred B and mitochondrial mutants MSC3, 12 and 16.

Gene	Line	Average Fold-change	S.E.	t-test
gadph ^{n}	Line B	0.89	0.06	CONTROL
gadph ^{n}	MSC3	0.86	0.10	0.4059
gadph ^{n}	MSC12	0.79	0.05	0.1267
gadph ^{n}	MSC16	0.83	0.05	0.2371
actin3 ^{n}	Line B	1.00	0.06	CONTROL
actin3 ^{n}	MSC3	1.21	0.08	0.0574
actin3 ^{n}	MSC12	1.05	0.04	0.2899
actin3 ^{n}	MSC16	1.08	0.08	0.2600
nad9	Line B	1.00	0.02	CONTROL
nad9	MSC3	1.38	0.22	0.0782
nad9	MSC12	2.60	0.25	0.0016*
nad9	MSC16	1.88	0.16	0.0031*
cob	Line B	1.00	0.02	CONTROL
cob	MSC3	1.29	0.05	0.0034*
cob	MSC12	1.47	0.20	0.0400*
cob	MSC16	1.32	0.08	0.0096*
rps7	Line B	1.02	0.15	CONTROL
rps7	MSC3	0.92	0.16	0.3371
rps7	MSC12	0.12	0.04	0.0021*
rps7	MSC16	0.12	0.05	0.0023*
ccmFc	Line B	1.00	0.04	CONTROL
ccmFc	MSC3	1.31	0.16	0.0715
ccmFc	MSC12	2.09	0.32	0.0143*

ccmFc	MSC16	2.38	0.75	0.0716
nad7	Line B	1.00	0.07	CONTROL
nad7	MSC3	1.04	0.12	0.4124
nad7	MSC12	1.23	0.18	0.1535
nad7	MSC16	1.08	0.12	0.2998
ccmB	Line B	1.05	0.23	CONTROL
ccmB	MSC3	1.30	0.21	0.2366
ccmB	MSC12	1.68	0.17	0.0482*
ccmB	MSC16	1.59	0.28	0.1039
atp1	Line B	1.03	0.16	CONTROL
atp1	MSC3	1.16	0.14	0.2794
atp1	MSC12	2.22	0.26	0.0089*
atp1	MSC16	2.42	0.24	0.0042*
cox1	Line B	1.01	0.10	CONTROL
cox1	MSC3	1.20	0.25	0.2568
cox1	MSC12	1.71	0.17	0.0116*
cox1	MSC16	2.04	0.28	0.0127*
nad6	Line B	1.01	0.10	CONTROL
nad6	MSC3	1.27	0.14	0.0985
nad6	MSC12	2.15	0.17	0.0023*
nad6	MSC16	2.38	0.25	0.0039*
ubqc [n]	Line B	1.00	0.00	CONTROL
ubqc [n]	MSC3	1.11	0.12	0.2041
ubqc [n]	MSC12	1.05	0.09	0.2886
ubqc [n]	MSC16	1.03	0.10	0.3820
sdh3	Line B	1.04	0.25	CONTROL

sdh3	MSC3	1.23	0.07	0.2100
sdh3	MSC12	2.09	0.09	0.0048*
sdh3	MSC16	2.16	0.36	0.0265*
nad3	Line B	1.02	0.16	CONTROL
nad3	MSC3	1.43	0.16	0.0741
nad3	MSC12	2.00	0.08	0.0029*
nad3	MSC16	1.88	0.33	0.0402*
rrn5	Line B	1.01	0.12	CONTROL
rrn5	MSC3	1.05	0.10	0.4057
rrn5	MSC12	1.17	0.00	0.1311
rrn5	MSC16	1.26	0.05	0.0683
rpl2	Line B	1.00	0.04	CONTROL
rpl2	MSC3	1.00	0.08	0.4799
rpl2	MSC12	1.86	0.07	0.0003*
rpl2	MSC16	2.10	0.12	0.0006*
rps3	Line B	1.01	0.12	CONTROL
rps3	MSC3	1.40	0.16	0.0660
rps3	MSC12	2.31	0.20	0.0025*
rps3	MSC16	2.53	0.20	0.0015*
rpl5	Line B	1.00	0.07	CONTROL
rpl5	MSC3	1.38	0.19	0.0710
rpl5	MSC12	2.05	0.32	0.0171*
rpl5	MSC16	2.86	0.34	0.0030*
nad5	Line B	1.03	0.19	CONTROL
nad5	MSC3	0.36	0.11	0.0194*
nad5	MSC12	1.85	0.39	0.0661

nad5	MSC16	1.47	0.20	0.0931
atp4	Line B	1.03	0.19	CONTROL
atp4	MSC3	0.43	0.04	0.0182*
atp4	MSC12	1.69	0.31	0.0724
atp4	MSC16	1.88	0.19	0.0170*
rrnS	Line B	1.01	0.12	CONTROL
rrnS	MSC3	1.06	0.11	0.4046
rrnS	MSC12	1.29	0.06	0.0556
rrnS	MSC16	1.39	0.11	0.0432*
aox ^{nm}	Line B	1.00	0.07	CONTROL
aox ^{nm}	MSC3	1.21	0.22	0.2082
aox ^{nm}	MSC12	1.00	0.07	0.4992
aox ^{nm}	MSC16	1.24	0.10	0.0640
porin ^{nm}	Line B	1.16	0.47	CONTROL
porin ^{nm}	MSC3	34.74	33.31	0.1853
porin ^{nm}	MSC12	0.80	0.20	0.2589
porin ^{nm}	MSC16	2.34	1.03	0.1788
cox2	Line B	1.00	0.07	CONTROL
cox2	MSC3	1.12	0.30	0.3596
cox2	MSC12	1.79	0.14	0.0040*
cox2	MSC16	1.95	0.04	0.0002*
rps13	Line B	1.04	0.21	CONTROL
rps13	MSC3	1.33	0.23	0.1968
rps13	MSC12	2.19	0.00	0.0025*
rps13	MSC16	2.05	0.30	0.0253*
mttb	Line B	1.06	0.26	CONTROL

mttb	MSC3	1.42	0.11	0.1383
mttb	MSC12	2.46	0.06	0.0033*
mttb	MSC16	2.26	0.23	0.0130*
rps10	Line B	1.00	0.08	CONTROL
rps10	MSC3	1.12	0.05	0.1431
rps10	MSC12	2.02	0.24	0.0080*
rps10	MSC16	2.25	0.13	0.0007*
atp8	Line B	1.00	0.04	CONTROL
atp8	MSC3	1.15	0.09	0.0998
atp8	MSC12	1.55	0.04	0.0003*
atp8	MSC16	1.82	0.04	0.0001*
rrnL	Line B	1.00	0.04	CONTROL
rrnL	MSC3	1.25	0.15	0.0952
rrnL	MSC12	3.03	0.68	0.0207*
rrnL	MSC16	2.25	0.53	0.0400*

* Significant difference between an MSC mutant for a given mitochondrial or nuclear gene compared to wild-type B at $\alpha = 0.05$, Student's t-test.

[n] Nuclear-encoded gene with no function in the mitochondria.

[nm] Nuclear-encoded mitochondrially targeted gene.

Table S3 Fold change in transcript levels of mitochondrial and nuclear genes from wild-type inbred B and mitochondrial mutants MSC3, 12 and 16.

Gene	Line	Average Fold-change	S.E.	t-test
rps7	Line B	1.12	0.18	CONTROL
rps7	MSC3	2.32	0.34	0.0044*
rps7	MSC12	0.21	0.07	0.0005*
rps7	MSC16	0.36	0.08	0.0016*
rrn5	Line B	1.05	0.12	CONTROL
rrn5	MSC3	1.67	0.49	0.1246
rrn5	MSC12	1.15	0.19	0.3181
rrn5	MSC16	1.16	0.16	0.2837
rpl2	Line B	1.09	0.37	CONTROL
rpl2	MSC3	1.76	0.47	0.0148*
rpl2	MSC12	2.00	0.29	0.0087*
rpl2	MSC16	2.00	0.31	0.0118*
nad3	Line B	1.31	0.32	CONTROL
nad3	MSC3	2.06	0.46	0.1028
nad3	MSC12	2.92	0.46	0.0041*
nad3	MSC16	4.75	1.14	0.0087*
sdh3	Line B	1.08	0.16	CONTROL
sdh3	MSC3	2.08	0.48	0.0395*
sdh3	MSC12	2.05	0.43	0.0256*
sdh3	MSC16	1.85	0.42	0.0583
nad6	Line B	1.23	0.28	CONTROL
nad6	MSC3	1.55	0.31	0.2295
nad6	MSC12	2.54	0.64	0.0379*

nad6	MSC16	2.57	0.66	0.0435*
ubqc [n]	Line B	1.09	0.35	CONTROL
ubqc [n]	MSC3	1.51	0.80	0.1613
ubqc [n]	MSC12	1.11	0.21	0.4583
ubqc [n]	MSC16	1.36	0.25	0.1873
nad9	Line B	1.43	0.48	CONTROL
nad9	MSC3	2.13	0.40	0.1391
nad9	MSC12	4.40	1.11	0.0122*
nad9	MSC16	5.21	1.71	0.0309*
cob	Line B	1.19	0.25	CONTROL
cob	MSC3	1.92	0.26	0.0301*
cob	MSC12	2.13	0.34	0.0156*
cob	MSC16	3.13	0.71	0.0142*
actin3 [n]	Line B	3.46	2.54	CONTROL
actin3 [n]	MSC3	3.15	0.86	0.4545
actin3 [n]	MSC12	2.88	1.41	0.4222
actin3 [n]	MSC16	3.99	0.87	0.4244
rps10	Line B	1.19	0.50	CONTROL
rps10	MSC3	1.90	1.32	0.1541
rps10	MSC12	1.89	0.41	0.0790
rps10	MSC16	2.64	0.53	0.0143*
rps3	Line B	1.09	0.17	CONTROL
rps3	MSC3	1.21	0.20	0.3276
rps3	MSC12	2.04	0.34	0.0097*
rps3	MSC16	1.93	0.37	0.0328*
mttb	Line B	1.07	0.16	CONTROL

mttb	MSC3	1.57	0.34	0.1084
mttb	MSC12	2.01	0.32	0.0067*
mttb	MSC16	1.82	0.24	0.0113*
atp4	Line B	1.03	0.09	CONTROL
atp4	MSC3	0.47	0.07	0.0001*
atp4	MSC12	1.12	0.19	0.3112
atp4	MSC16	1.01	0.14	0.4523
cox2	Line B	1.03	0.18	CONTROL
cox2	MSC3	1.12	0.24	0.2669
cox2	MSC12	1.25	0.15	0.1114
cox2	MSC16	1.32	0.18	0.0917
aox ^{nm}	Line B	1.03	0.10	CONTROL
aox ^{nm}	MSC3	1.94	0.22	0.0017*
aox ^{nm}	MSC12	2.33	0.27	0.0003*
aox ^{nm}	MSC16	2.66	0.35	0.0007*
rrnS	Line B	1.06	0.13	CONTROL
rrnS	MSC3	1.35	0.18	0.1090
rrnS	MSC12	0.73	0.12	0.0383*
rrnS	MSC16	0.82	0.11	0.0905
rps13	Line B	1.10	0.17	CONTROL
rps13	MSC3	1.35	0.11	0.1150
rps13	MSC12	2.85	0.39	0.0003*
rps13	MSC16	2.82	0.71	0.0213*
rpl5	Line B	1.09	0.39	CONTROL
rpl5	MSC3	1.81	0.45	0.0112*
rpl5	MSC12	2.27	0.57	0.0396*

rpl5	MSC16	2.05	0.23	0.0026*
atp8	Line B	1.02	0.08	CONTROL
atp8	MSC3	1.09	0.23	0.3999
atp8	MSC12	1.33	0.23	0.0916
atp8	MSC16	1.45	0.22	0.0504
nad5	Line B	1.13	0.22	CONTROL
nad5	MSC3	0.14	0.02	0.0011*
nad5	MSC12	1.28	0.34	0.3535
nad5	MSC16	1.16	0.27	0.4686
rrnL	Line B	1.06	0.12	CONTROL
rrnL	MSC3	1.15	0.21	0.3519
rrnL	MSC12	1.19	0.30	0.3461
rrnL	MSC16	1.51	0.28	0.0843
cox1	Line B	1.13	0.41	CONTROL
cox1	MSC3	1.58	0.68	0.1262
cox1	MSC12	1.85	0.58	0.1347
cox1	MSC16	3.07	1.03	0.0500*
atp1	Line B	1.11	0.19	CONTROL
atp1	MSC3	2.05	0.24	0.0039*
atp1	MSC12	1.77	0.57	0.1510
atp1	MSC16	1.91	0.50	0.0849
nad1	Line B	1.03	0.08	CONTROL
nad1	MSC3	0.95	0.14	0.3251
nad1	MSC12	1.08	0.32	0.4367
nad1	MSC16	1.18	0.31	0.3286
nad7	Line B	1.05	0.11	CONTROL

nad7	MSC3	1.16	0.16	0.2910
nad7	MSC12	1.06	0.25	0.4889
nad7	MSC16	1.19	0.25	0.3109
ccmB	Line B	1.10	0.31	CONTROL
ccmB	MSC3	2.15	1.41	0.0784
ccmB	MSC12	1.55	0.19	0.0400*
ccmB	MSC16	2.30	0.40	0.0090*
porin [n]	Line B	1.08	0.15	CONTROL
porin [n]	MSC3	1.01	0.19	0.3941
porin [n]	MSC12	0.80	0.23	0.1624
porin [n]	MSC16	0.87	0.16	0.1690
ccmFc	Line B	1.08	0.15	CONTROL
ccmFc	MSC3	1.34	0.22	0.1781
ccmFc	MSC12	2.81	0.71	0.0176*
ccmFc	MSC16	2.34	0.47	0.0145*
gadph [n]	Line B	1.03	0.08	CONTROL
gadph [n]	MSC3	1.32	0.17	0.0829
gadph [n]	MSC12	1.17	0.17	0.2137
gadph [n]	MSC16	1.14	0.26	0.3495

* Significant difference between an MSC mutant for a given mitochondrial or nuclear gene compared to wild-type B at $\alpha = 0.05$, Student's t-test.

[n] Nuclear-encoded gene with no function in the mitochondria.

[nm] Nuclear-encoded mitochondrially targeted gene.

Table S4 Primer sequences that were used for quantitative-PCR.

Location	Gene	Forward Primer	Reverse Primer
Nuclear	*gadph*	AGGAAGAGTCCGAGGGAAAA	ACGAGGATGTGAAGCCAAAC
	aox2	GCACCATCAGCCCAAAAC	AGCATCCCTCCAACCATTC
	ubqc	CACAGCTCGATATTGGACAGA	AATTTTCATCCCAACCCACA
	actin3	CCAAGGCGAATAGAGAGAAAA	GCAACATACATAGCGGGAGTG
Complex I	*nad9*	ACAAACCAGTGCAGACGAAG	CCGGATGATTGATGGAAGAA
	nad7	GCAGCACCTTACGATGTTCA	TGTCGCATCTCTTCGATACG
	nad6	TCCGTCTGGTTTTTGCTTTC	GTGGTTCTGTCGTCCTCCTC
	nad3	CACTCGGTCTTCCTTTTCCA	AACGACTTCTGGCATCACC
	nad5 ex4	AGGATATTAGGGGAAGCAGTGAG	CCAAGAAGATAGAGAGTCCCACA
Complex II	*sdh3*	TGGGTTTGATTTGCTTCACC	CGACGGATATTAGGATGAGCTTT
Complex III	*cob*	CTTGGTGAATAGGGCGAAAA	TGACAAAGCGGGAGGTGTAG
Complex IV	*cox1*	TTCCCATGCATTTCTTAGGG	AACGACGAATCCCAACTACG
	cox2	GATCTCAAGACGCAGCAACA	CATAAAGCGCGAACCAAGA
	ccmFc	GCCTCATTTCCTTTCCTTCC	TCACGCTTCGCTGAACTATG
	ccmB	CGGAATGGATCGGTTAAACA	AAGAGCCGAACGAGAATGAA
Complex V	*atp1*	TGAAACACAAGCTGGAGACG	ACACGACTGACGGATAAGCC
	atp4	GCAGCAATTTCCCAATCCT	TGCACTGTCTTTTCGCACTT
	atp8	TGCCTTTTCTTCTTGACTTTCT	TCCTTGCTCCGTATGTTCTTC
Ribosome LU	*rpl2*	AGCCAAGAGGGGAGAGCA	CCGAGAAGAACGATTAGGG
	rpl5	GCCCGGAGAATTTAAGAAAGA	TGACCTAGCACGAGAAAGCA
	rrnL	GTCAGCGAGAAAATGGGAAC	CGGGTCAAATAGGAAGAACGA
Ribosome SU	*rrn5*	AGCGATCGACGTGAAAACAC	TCCTTCATTACTAGCCGC
	rrnS	TGCGCTTTAGTTTGATTGCT	AGTCTGAGGACCCGTTTTGG
	rps7	TGACGATCCCTGGCTACAA	ATGGTTGAGGCCGTAGAGAA
	rps13	GTCTGCTCGTTCTCCCCTCT	AAAAAGCCATTCAGGTTCGTT
	rps10	GGAAGATTGGATTGCCTGAA	ATGCCTTTCTGTTTTTATGACC
	rps3	GGAGCGAATACACAGGGAAG	CGAAGAAAGGAAAGAGCGAGA
