# Supplementary material for: The Mosaic Mutants of Cucumber: A Method to Produce Knock-Downs of Mitochondrial Transcripts
Source: G3 (Bethesda). 2015 Apr 14;5(6):1211–21. doi: 10.1534/g3.115.017053 (PMC4478549; doi:10.1534/g3.115.017053)
Supplement: Supporting Information [file supp_g3.115.017053_TableS1.pdf]

Table S1 Low-coverage regions in the mt DNA of the mitochondrial mutant MSC3, 12, and 16 as compared to the progenitor, wild-type inbred B.

Mutant Line	Region (bp) Start	End	Size (bp)	Chromosome*	Annotation
MSC3	392,242	441,105	48,863	MC	*nad5ex4* (NADH dehydrogenase subunit 5 C-terminus); trnW-CCA (tRNA-Trp); *atp4* (ATPase subunit 4) and chloroplast-like DNA
	585,075	636,619	51,544	MC	
	1	31,162	31,161	83.8 kb	
		Tota	131,568	7.8%	
MSC12	149,606	161,705	12,099	MC	
	195,676	267,406	71,730	MC	*rps7* (ribosomal protein S7)
	347,550	362,669	15,119	MC	JLV5-Del (Lilly *et al.* 2001) was identified in MSC16)
	567,690	568,518	828	MC	
	572,554	622,942	50,388	MC	
	911,926	917,574	5,648	MC	
	22,564	25,406	2,842	44.8 kb	
	1	83,817	83,816	83.8 kb	
		Total	242,470	14.4%	
MSC16	149,606	161,706	12,100	MC	
	195,676	283,618	87,942	MC	*rps7* (ribosomal protein S7)
	347,550	362,669	15,119	MC	JLV5-Del (Lilly *et al.* 2001)
	567,711	568,451	740	MC	
	572,554	622,942	50,388	MC	
	911,926	917,574	5,648	MC	
	22,564	25,270	2,706	44.8 kb	
	1	83,817	83,816	83.8 kb	
		Total	258,459	15.3%	

MC = Master chromosome of 1.6 megabases (Mb).

44.8 kb = Extra-chromosome of 44.8 kb

83.8 kb = Extra-chromosome of 83.8 kb

*All locations are based on the mitochondrial sequence of Alverson *et al.* (2011).
