# Supplementary material for: The Mosaic Mutants of Cucumber: A Method to Produce Knock-Downs of Mitochondrial Transcripts
Source: G3 (Bethesda). 2015 Apr 14;5(6):1211–21. doi: 10.1534/g3.115.017053 (PMC4478549; doi:10.1534/g3.115.017053)
Supplement: Supporting Information [file supp_g3.115.017053_TableS2.pdf]

Table S2 Fold change of copy number of mitochondrial and nuclear genes in wild-type inbred B and mitochondrial mutants MSC3, 12 and 16.

Gene	Line	Average Fold-change	S.E.	t-test
gadph ^{n}	Line B	0.89	0.06	CONTROL
gadph ^{n}	MSC3	0.86	0.10	0.4059
gadph ^{n}	MSC12	0.79	0.05	0.1267
gadph ^{n}	MSC16	0.83	0.05	0.2371
actin3 ^{n}	Line B	1.00	0.06	CONTROL
actin3 ^{n}	MSC3	1.21	0.08	0.0574
actin3 ^{n}	MSC12	1.05	0.04	0.2899
actin3 ^{n}	MSC16	1.08	0.08	0.2600
nad9	Line B	1.00	0.02	CONTROL
nad9	MSC3	1.38	0.22	0.0782
nad9	MSC12	2.60	0.25	0.0016*
nad9	MSC16	1.88	0.16	0.0031*
cob	Line B	1.00	0.02	CONTROL
cob	MSC3	1.29	0.05	0.0034*
cob	MSC12	1.47	0.20	0.0400*
cob	MSC16	1.32	0.08	0.0096*
rps7	Line B	1.02	0.15	CONTROL
rps7	MSC3	0.92	0.16	0.3371
rps7	MSC12	0.12	0.04	0.0021*
rps7	MSC16	0.12	0.05	0.0023*
ccmFc	Line B	1.00	0.04	CONTROL
ccmFc	MSC3	1.31	0.16	0.0715
ccmFc	MSC12	2.09	0.32	0.0143*

ccmFc	MSC16	2.38	0.75	0.0716
nad7	Line B	1.00	0.07	CONTROL
nad7	MSC3	1.04	0.12	0.4124
nad7	MSC12	1.23	0.18	0.1535
nad7	MSC16	1.08	0.12	0.2998
ccmB	Line B	1.05	0.23	CONTROL
ccmB	MSC3	1.30	0.21	0.2366
ccmB	MSC12	1.68	0.17	0.0482*
ccmB	MSC16	1.59	0.28	0.1039
atp1	Line B	1.03	0.16	CONTROL
atp1	MSC3	1.16	0.14	0.2794
atp1	MSC12	2.22	0.26	0.0089*
atp1	MSC16	2.42	0.24	0.0042*
cox1	Line B	1.01	0.10	CONTROL
cox1	MSC3	1.20	0.25	0.2568
cox1	MSC12	1.71	0.17	0.0116*
cox1	MSC16	2.04	0.28	0.0127*
nad6	Line B	1.01	0.10	CONTROL
nad6	MSC3	1.27	0.14	0.0985
nad6	MSC12	2.15	0.17	0.0023*
nad6	MSC16	2.38	0.25	0.0039*
ubqc [n]	Line B	1.00	0.00	CONTROL
ubqc [n]	MSC3	1.11	0.12	0.2041
ubqc [n]	MSC12	1.05	0.09	0.2886
ubqc [n]	MSC16	1.03	0.10	0.3820
sdh3	Line B	1.04	0.25	CONTROL

sdh3	MSC3	1.23	0.07	0.2100
sdh3	MSC12	2.09	0.09	0.0048*
sdh3	MSC16	2.16	0.36	0.0265*
nad3	Line B	1.02	0.16	CONTROL
nad3	MSC3	1.43	0.16	0.0741
nad3	MSC12	2.00	0.08	0.0029*
nad3	MSC16	1.88	0.33	0.0402*
rrn5	Line B	1.01	0.12	CONTROL
rrn5	MSC3	1.05	0.10	0.4057
rrn5	MSC12	1.17	0.00	0.1311
rrn5	MSC16	1.26	0.05	0.0683
rpl2	Line B	1.00	0.04	CONTROL
rpl2	MSC3	1.00	0.08	0.4799
rpl2	MSC12	1.86	0.07	0.0003*
rpl2	MSC16	2.10	0.12	0.0006*
rps3	Line B	1.01	0.12	CONTROL
rps3	MSC3	1.40	0.16	0.0660
rps3	MSC12	2.31	0.20	0.0025*
rps3	MSC16	2.53	0.20	0.0015*
rpl5	Line B	1.00	0.07	CONTROL
rpl5	MSC3	1.38	0.19	0.0710
rpl5	MSC12	2.05	0.32	0.0171*
rpl5	MSC16	2.86	0.34	0.0030*
nad5	Line B	1.03	0.19	CONTROL
nad5	MSC3	0.36	0.11	0.0194*
nad5	MSC12	1.85	0.39	0.0661

nad5	MSC16	1.47	0.20	0.0931
atp4	Line B	1.03	0.19	CONTROL
atp4	MSC3	0.43	0.04	0.0182*
atp4	MSC12	1.69	0.31	0.0724
atp4	MSC16	1.88	0.19	0.0170*
rrnS	Line B	1.01	0.12	CONTROL
rrnS	MSC3	1.06	0.11	0.4046
rrnS	MSC12	1.29	0.06	0.0556
rrnS	MSC16	1.39	0.11	0.0432*
aox ^{nm}	Line B	1.00	0.07	CONTROL
aox ^{nm}	MSC3	1.21	0.22	0.2082
aox ^{nm}	MSC12	1.00	0.07	0.4992
aox ^{nm}	MSC16	1.24	0.10	0.0640
porin ^{nm}	Line B	1.16	0.47	CONTROL
porin ^{nm}	MSC3	34.74	33.31	0.1853
porin ^{nm}	MSC12	0.80	0.20	0.2589
porin ^{nm}	MSC16	2.34	1.03	0.1788
cox2	Line B	1.00	0.07	CONTROL
cox2	MSC3	1.12	0.30	0.3596
cox2	MSC12	1.79	0.14	0.0040*
cox2	MSC16	1.95	0.04	0.0002*
rps13	Line B	1.04	0.21	CONTROL
rps13	MSC3	1.33	0.23	0.1968
rps13	MSC12	2.19	0.00	0.0025*
rps13	MSC16	2.05	0.30	0.0253*
mttb	Line B	1.06	0.26	CONTROL

mttb	MSC3	1.42	0.11	0.1383
mttb	MSC12	2.46	0.06	0.0033*
mttb	MSC16	2.26	0.23	0.0130*
rps10	Line B	1.00	0.08	CONTROL
rps10	MSC3	1.12	0.05	0.1431
rps10	MSC12	2.02	0.24	0.0080*
rps10	MSC16	2.25	0.13	0.0007*
atp8	Line B	1.00	0.04	CONTROL
atp8	MSC3	1.15	0.09	0.0998
atp8	MSC12	1.55	0.04	0.0003*
atp8	MSC16	1.82	0.04	0.0001*
rrnL	Line B	1.00	0.04	CONTROL
rrnL	MSC3	1.25	0.15	0.0952
rrnL	MSC12	3.03	0.68	0.0207*
rrnL	MSC16	2.25	0.53	0.0400*

* Significant difference between an MSC mutant for a given mitochondrial or nuclear gene compared to wild-type B at $\alpha = 0.05$, Student's t-test.

[n] Nuclear-encoded gene with no function in the mitochondria.

[nm] Nuclear-encoded mitochondrially targeted gene.
