# Supplementary material for: The Mosaic Mutants of Cucumber: A Method to Produce Knock-Downs of Mitochondrial Transcripts
Source: G3 (Bethesda). 2015 Apr 14;5(6):1211–21. doi: 10.1534/g3.115.017053 (PMC4478549; doi:10.1534/g3.115.017053)
Supplement: Supporting Information [file supp_g3.115.017053_TableS3.pdf]

Table S3 Fold change in transcript levels of mitochondrial and nuclear genes from wild-type inbred B and mitochondrial mutants MSC3, 12 and 16.

Gene	Line	Average Fold-change	S.E.	t-test
rps7	Line B	1.12	0.18	CONTROL
rps7	MSC3	2.32	0.34	0.0044*
rps7	MSC12	0.21	0.07	0.0005*
rps7	MSC16	0.36	0.08	0.0016*
rrn5	Line B	1.05	0.12	CONTROL
rrn5	MSC3	1.67	0.49	0.1246
rrn5	MSC12	1.15	0.19	0.3181
rrn5	MSC16	1.16	0.16	0.2837
rpl2	Line B	1.09	0.37	CONTROL
rpl2	MSC3	1.76	0.47	0.0148*
rpl2	MSC12	2.00	0.29	0.0087*
rpl2	MSC16	2.00	0.31	0.0118*
nad3	Line B	1.31	0.32	CONTROL
nad3	MSC3	2.06	0.46	0.1028
nad3	MSC12	2.92	0.46	0.0041*
nad3	MSC16	4.75	1.14	0.0087*
sdh3	Line B	1.08	0.16	CONTROL
sdh3	MSC3	2.08	0.48	0.0395*
sdh3	MSC12	2.05	0.43	0.0256*
sdh3	MSC16	1.85	0.42	0.0583
nad6	Line B	1.23	0.28	CONTROL
nad6	MSC3	1.55	0.31	0.2295
nad6	MSC12	2.54	0.64	0.0379*

nad6	MSC16	2.57	0.66	0.0435*
ubqc [n]	Line B	1.09	0.35	CONTROL
ubqc [n]	MSC3	1.51	0.80	0.1613
ubqc [n]	MSC12	1.11	0.21	0.4583
ubqc [n]	MSC16	1.36	0.25	0.1873
nad9	Line B	1.43	0.48	CONTROL
nad9	MSC3	2.13	0.40	0.1391
nad9	MSC12	4.40	1.11	0.0122*
nad9	MSC16	5.21	1.71	0.0309*
cob	Line B	1.19	0.25	CONTROL
cob	MSC3	1.92	0.26	0.0301*
cob	MSC12	2.13	0.34	0.0156*
cob	MSC16	3.13	0.71	0.0142*
actin3 [n]	Line B	3.46	2.54	CONTROL
actin3 [n]	MSC3	3.15	0.86	0.4545
actin3 [n]	MSC12	2.88	1.41	0.4222
actin3 [n]	MSC16	3.99	0.87	0.4244
rps10	Line B	1.19	0.50	CONTROL
rps10	MSC3	1.90	1.32	0.1541
rps10	MSC12	1.89	0.41	0.0790
rps10	MSC16	2.64	0.53	0.0143*
rps3	Line B	1.09	0.17	CONTROL
rps3	MSC3	1.21	0.20	0.3276
rps3	MSC12	2.04	0.34	0.0097*
rps3	MSC16	1.93	0.37	0.0328*
mttb	Line B	1.07	0.16	CONTROL

mttb	MSC3	1.57	0.34	0.1084
mttb	MSC12	2.01	0.32	0.0067*
mttb	MSC16	1.82	0.24	0.0113*
atp4	Line B	1.03	0.09	CONTROL
atp4	MSC3	0.47	0.07	0.0001*
atp4	MSC12	1.12	0.19	0.3112
atp4	MSC16	1.01	0.14	0.4523
cox2	Line B	1.03	0.18	CONTROL
cox2	MSC3	1.12	0.24	0.2669
cox2	MSC12	1.25	0.15	0.1114
cox2	MSC16	1.32	0.18	0.0917
aox^{nm}	Line B	1.03	0.10	CONTROL
aox^{nm}	MSC3	1.94	0.22	0.0017*
aox^{nm}	MSC12	2.33	0.27	0.0003*
aox^{nm}	MSC16	2.66	0.35	0.0007*
rrnS	Line B	1.06	0.13	CONTROL
rrnS	MSC3	1.35	0.18	0.1090
rrnS	MSC12	0.73	0.12	0.0383*
rrnS	MSC16	0.82	0.11	0.0905
rps13	Line B	1.10	0.17	CONTROL
rps13	MSC3	1.35	0.11	0.1150
rps13	MSC12	2.85	0.39	0.0003*
rps13	MSC16	2.82	0.71	0.0213*
rpl5	Line B	1.09	0.39	CONTROL
rpl5	MSC3	1.81	0.45	0.0112*
rpl5	MSC12	2.27	0.57	0.0396*

rpl5	MSC16	2.05	0.23	0.0026*
atp8	Line B	1.02	0.08	CONTROL
atp8	MSC3	1.09	0.23	0.3999
atp8	MSC12	1.33	0.23	0.0916
atp8	MSC16	1.45	0.22	0.0504
nad5	Line B	1.13	0.22	CONTROL
nad5	MSC3	0.14	0.02	0.0011*
nad5	MSC12	1.28	0.34	0.3535
nad5	MSC16	1.16	0.27	0.4686
rrnL	Line B	1.06	0.12	CONTROL
rrnL	MSC3	1.15	0.21	0.3519
rrnL	MSC12	1.19	0.30	0.3461
rrnL	MSC16	1.51	0.28	0.0843
cox1	Line B	1.13	0.41	CONTROL
cox1	MSC3	1.58	0.68	0.1262
cox1	MSC12	1.85	0.58	0.1347
cox1	MSC16	3.07	1.03	0.0500*
atp1	Line B	1.11	0.19	CONTROL
atp1	MSC3	2.05	0.24	0.0039*
atp1	MSC12	1.77	0.57	0.1510
atp1	MSC16	1.91	0.50	0.0849
nad1	Line B	1.03	0.08	CONTROL
nad1	MSC3	0.95	0.14	0.3251
nad1	MSC12	1.08	0.32	0.4367
nad1	MSC16	1.18	0.31	0.3286
nad7	Line B	1.05	0.11	CONTROL

nad7	MSC3	1.16	0.16	0.2910
nad7	MSC12	1.06	0.25	0.4889
nad7	MSC16	1.19	0.25	0.3109
ccmB	Line B	1.10	0.31	CONTROL
ccmB	MSC3	2.15	1.41	0.0784
ccmB	MSC12	1.55	0.19	0.0400*
ccmB	MSC16	2.30	0.40	0.0090*
porin ^{n}	Line B	1.08	0.15	CONTROL
porin ^{n}	MSC3	1.01	0.19	0.3941
porin ^{n}	MSC12	0.80	0.23	0.1624
porin ^{n}	MSC16	0.87	0.16	0.1690
ccmFc	Line B	1.08	0.15	CONTROL
ccmFc	MSC3	1.34	0.22	0.1781
ccmFc	MSC12	2.81	0.71	0.0176*
ccmFc	MSC16	2.34	0.47	0.0145*
gadph ^{n}	Line B	1.03	0.08	CONTROL
gadph ^{n}	MSC3	1.32	0.17	0.0829
gadph ^{n}	MSC12	1.17	0.17	0.2137
gadph ^{n}	MSC16	1.14	0.26	0.3495

* Significant difference between an MSC mutant for a given mitochondrial or nuclear gene compared to wild-type B at $\alpha = 0.05$, Student's t-test.

^{n} Nuclear-encoded gene with no function in the mitochondria.

^{nm} Nuclear-encoded mitochondrially targeted gene.
