# Supplementary material for: The Mosaic Mutants of Cucumber: A Method to Produce Knock-Downs of Mitochondrial Transcripts
Source: G3 (Bethesda). 2015 Apr 14;5(6):1211–21. doi: 10.1534/g3.115.017053 (PMC4478549; doi:10.1534/g3.115.017053)
Supplement: Supporting Information [file supp_g3.115.017053_TableS4.pdf]

Table S4   Primer sequences that were used for quantitative-PCR.

Location	Gene	Forward Primer	Reverse Primer
Nuclear	*gadph*	AGGAAGAGTCCGAGGGAAAA	ACGAGGATGTGAAGCCAAAC
	aox2	GCACCATCAGCCCAAAAC	AGCATCCCTCCAACCATTC
	ubqc	CACAGCTCGATATTGGACAGA	AATTTTCATCCCAACCCACA
	actin3	CCAAGGCGAATAGAGAGAAAA	GCAACATACATAGCGGGAGTG
Complex I	*nad9*	ACAAACCAGTGCAGACGAAG	CCGGATGATTGATGGAAGAA
	nad7	GCAGCACCTTACGATGTTCA	TGTCGCATCTCTTCGATACG
	nad6	TCCGTCTGGTTTTTGCTTTC	GTGGTTCTGTCGTCCTCCTC
	nad3	CACTCGGTCTTCCTTTTCCA	AACGACTTCTGGCATCACC
	nad5 ex4	AGGATATTAGGGGAAGCAGTGAG	CCAAGAAGATAGAGAGTCCCACA
Complex II	*sdh3*	TGGGTTTGATTTGCTTCACC	CGACGGATATTAGGATGAGCTTT
Complex III	*cob*	CTTGGTGAATAGGGCGAAAA	TGACAAAGCGGGAGGTGTAG
Complex IV	*cox1*	TTCCCATGCATTTCTTAGGG	AACGACGAATCCCAACTACG
	cox2	GATCTCAAGACGCAGCAACA	CATAAAGCGCGAACCAAGA
	ccmFc	GCCTCATTTCCTTTCCTTCC	TCACGCTTCGCTGAACTATG
	ccmB	CGGAATGGATCGGTTAAACA	AAGAGCCGAACGAGAATGAA
Complex V	*atp1*	TGAAACACAAGCTGGAGACG	ACACGACTGACGGATAAGCC
	atp4	GCAGCAATTTCCCAATCCT	TGCACTGTCTTTTCGCACTT
	atp8	TGCCTTTTCTTCTTGACTTTCT	TCCTTGCTCCGTATGTTCTTC
Ribosome LU	*rpl2*	AGCCAAGAGGGGAGAGCA	CCGAGAAGAACGATTAGGG
	rpl5	GCCCGGAGAATTTAAGAAAGA	TGACCTAGCACGAGAAAGCA
	rrnL	GTCAGCGAGAAAATGGGAAC	CGGGTCAAATAGGAAGAACGA
Ribosome SU	*rrn5*	AGCGATCGACGTGAAAACAC	TCCTTCATTACTAGCCGC
	rrnS	TGCGCTTTAGTTTGATTGCT	AGTCTGAGGACCCGTTTTGG
	rps7	TGACGATCCCTGGCTACAA	ATGGTTGAGGCCGTAGAGAA
	rps13	GTCTGCTCGTTCTCCCCTCT	AAAAAGCCATTCAGGTTCGTT
	rps10	GGAAGATTGGATTGCCTGAA	ATGCCTTTCTGTTTTTATGACC
	rps3	GGAGCGAATACACAGGGAAG	CGAAGAAAGGAAAGAGCGAGA
